# Targeted Sequencing of Large Genomic Regions with CATCH-Seq

**DOI:** 10.1371/journal.pone.0111756

**Published:** 2014-10-30

**Authors:** Kenneth Day, Jun Song, Devin Absher

**Affiliations:** HudsonAlpha Institute for Biotechnology, Huntsville, Alabama, United States of America; Bellvitge Biomedical Research Institute (IDIBELL), Spain

## Abstract

Current target enrichment systems for large-scale next-generation sequencing typically require synthetic oligonucleotides used as capture reagents to isolate sequences of interest. The majority of target enrichment reagents are focused on gene coding regions or promoters en masse. Here we introduce development of a customizable targeted capture system using biotinylated RNA probe baits transcribed from sheared bacterial artificial chromosome clone templates that enables capture of large, contiguous blocks of the genome for sequencing applications. This clone adapted template capture hybridization sequencing (CATCH-Seq) procedure can be used to capture both coding and non-coding regions of a gene, and resolve the boundaries of copy number variations within a genomic target site. Furthermore, libraries constructed with methylated adapters prior to solution hybridization also enable targeted bisulfite sequencing. We applied CATCH-Seq to diverse targets ranging in size from 125 kb to 3.5 Mb. Our approach provides a simple and cost effective alternative to other capture platforms because of template-based, enzymatic probe synthesis and the lack of oligonucleotide design costs. Given its similarity in procedure, CATCH-Seq can also be performed in parallel with commercial systems.

## Introduction

Costs for next generation sequencing technology and affiliated methods continue to fall for prospective whole genome sequencing of individuals. Along with growing improvements in sequencing technologies is a rapid expansion of our knowledge of human genetic variation and the impact of this sequence variation on human traits and diseases. High throughput sequencing is providing a foundation for both individualized patient therapies and newly developing programs in personalized medicine [1], [2]. However, individual whole genome sequencing currently remains expensive. The most practical alternative is to limit the sequencing per individual genome to select, meaningful loci by targeted enrichment to generate consistent high definition coverage around relevant regions [3].

Currently, solution hybridization based targeted capture for whole exome sequencing is perhaps the most consequential method for determination of sequence variation that directly affects human gene products [4], and similar capture methods can be used for targeting almost any region of the genome. Most large-scale targeted sequencing platforms rely upon the initial synthesis of tiled bait oligonucleotides of variable lengths across exons [5]. Synthesis of biotinylated RNA probe baits by in vitro transcription from oligonucleotide templates or direct use of biotinylated DNA oligonucleotide probe baits are used for solution hybridization capture of prepared sequencing libraries followed by binding to streptavidin beads and low to high stringency washes [6]. While these systems are cost-effective for the capture of large numbers of disparate targets, such as exons, this represents less than 2% of the human genome and excludes gene regulatory regions. Some commercially available products have emerged that emphasize capture of promoter regions containing CpG islands in the bait design based on cancer and tissue-specific differentially methylated regions [7]. Sequencing targets outside of exons or promoter regions requires customized synthesis of tiled oligonucleotides for the production of probe baits. As projects like ENCODE begin to identify critical regulatory regions of the human genome, the application of targeted sequencing to non-coding sequences has the potential to identify disease-related variation outside of the exome. Furthermore, there is increasing evidence for epigenetic influences in human diseases, and these epigenetic marks are often located in non-coding regions of the genome [8]. Additionally, the identification of regional structural variants, such as large deletions is difficult with exome sequencing data unless the variants overlap multiple exons of a given gene. Clearly, there is a growing need for targeted sequencing approaches that can interrogate both coding and non-coding elements around genes of interest.

A recent study demonstrated the custom capture of a 1 Mb contiguous site that encompassed the human dystrophin gene with use of densely designed oligo bait probes that spanned the entire region [9]. While sequencing of large contiguous regions in this manner is feasible, synthesis of custom probe baits remains a substantial added expense to sequencing costs, especially for small numbers of samples. Some techniques such as circularization-based methods using gap-fill padlock, MIPs or selector probe methods are feasible for targeted capture of specific regions, but also require design and synthesis of vast numbers of oligos, or careful restriction digest strategies [10]–[12]. Targeted amplification methods such as nested patch PCR carry the advantage of eliminating sequencing library construction along with high level multiplexing, but also requires oligonucleotides and is typically limited to relatively small composite targets [13].

Faster and more affordable alternatives are needed for targeted sequencing without the preliminary need of oligonucleotides to synthesize probe baits. Furthermore, many targets of interest in the genome are larger contiguous loci beyond the size of standard PCR amplicons, and may include coding and noncoding regions. Here we describe a new procedure using existing and commercially available genomic clones in clone adapted template capture hybridization sequencing (CATCH-Seq). Our method may be used all without oligonucleotide synthesis design to resolve copy number variation (CNV) boundaries, and, in combination with bisulfite treatment, to measure DNA methylation levels across large contiguous regions. Our method utilizes a simple approach for the targeted capture of any genomic region for which mapped clones exist, and is designed specifically for next-generation sequencing.

## Materials and Methods

### Illumina library construction

Concentrations of all input genomic DNAs were determined by Qubit high sensitivity double stranded DNA assays (Life Technologies), or by Picogreen assay in a 96-well 200 ul volume format using lambda DNA for a standard curve according to protocol (Life Technologies). Input genomic DNA quality was assessed by running 1–3 ul on a 1% agarose gel that contained 1× Sybr Green I dye (Life Technologies). Typical input DNA quantity for Illumina library construction ranged from 700 ng to 2.5 ug. Depending on the level of any DNA degradation before shearing, we often increased the quantity of input DNA. Genomic DNAs were adjusted to an 80 ul volume in water and placed into Covaris 96 microtube plates for shearing on an E210 E series focused ultrasonicator with installed intensifier at 4°C using fill level 6 and final run level 5. Program settings were 20% duty cycle, intensity 5, 200 cycles per burst, and treatment for 165 s. For repeat blocking optimization, we used K-562 cell line genomic DNA (ATCC CCL-243) obtained from the Myers Laboratory at HudsonAlpha Institute for Biotechnology for library construction.

Standard Illumina libraries were constructed using NEBNext End Repair, dA-Tailing and Quick Ligation modules (New England Biolabs). For cleanup steps following each enzymatic treatment, we used 1.8× reaction volume of SPRI Sera-Mag SpeedBeads (ThermoFisher) diluted 1∶40 in binding buffer containing 2.5M NaCl and 20% PEG-8000 as reported previously [14]. After DNA binding, beads were washed twice in 70% ethanol and dried for 10 min prior to elution in water. Oligos were synthesized and annealed to generate 12 methylated or 24 non-methylated inline barcoded adapter sets (Oblique Bio). We used 80–100 pmoles methylated or non-methylated adapters in the ligation step (one adapter per sample) depending on whether we used standard sequencing or bisulfite sequencing of the target enriched library and the quantity of input DNA. For lower input DNA, we adjusted the molar ratio of adapter. Final libraries were eluted in 40 ul water and library concentrations were determined by Qubit HS assay or by Picogreen using 1∶20 diluted libraries.

### Biotinylated probe synthesis

To prepare probes for target enrichment, we typically selected both Cal Tech human BAC library D (CTD) and RPCI human BAC library 11 (RP11) clones that covered targets of interest in order to avoid potential gaps due to any deletions in one clone. All clones were purchased from Life Technologies. BAC or fosmid DNAs were purified from 200 mL cultures grown for at 37°C 16 h that were inoculated from a single colony isolated from LB agar plates containing 25 ug/mL chloramphenicol using PureLink HiPure Plasmid Midi or Maxiprep kits (Life Technologies) with additional buffer provided in HiPure BAC buffer kits (Life Technologies). DNA concentration was determined by Qubit HS assay. Clone identity was verified by PCR or by HINDIII restriction digest of 1 ug DNA loaded on a 1.5% agarose gel containing 1× Sybr green (Life Technologies).

BAC DNAs were sheared under the same conditions as sample genomic DNAs. If multiple BACs were used, we created pools of individual BAC DNAs before shearing by calculating the percent of the total target size covered by each BAC multiplied by the total mass (typically 4 ug) of input pooled BAC DNA. Sheared BAC fragments were processed similarly to preparation of Illumina libraries except for use of T7 promoter-containing adapters (composed of two annealed oligos: TAC TAC TAA TAC GAC TCA CTA TAG GGT and CCC TAT AGT GAG TCG TAT TAG TAG TA) in the ligation step. Following cleanup of the ligation, 100–200 pmoles of T7 F oligo is annealed with T7-BAC fragments in a reaction containing 10× PCR buffer and 1 ul 50 mM MgCl_2_ in a 35 ul volume, heated to 95°C for 5 min, 50°C for 5 min, and cooled to 4°C. Following cleanup and elution into 36 ul water, three in vitro transcription (IVT) reactions each containing 12 ul template DNA was used for biotinylated RNA probe synthesis using a Megascript T7 kit (Ambion) according to manufacturer protocol including biotin-11-UTP (Life Technologies) for 1.5 h. Completed IVTs were DNAse treated according to protocol, 1 ul 0.5M EDTA was added, and then DNAse was heat inactivated for 10 min at 75°C. Reactions were pooled and a 2 ul aliquot was mixed with gel loading buffer (heated to 95°C for 2 min) and loaded on a 1.5% agarose gel with 10 ug/mL ethidium bromide to assess yield. Final pooled probe reactions were cleaned up using NucAway gel filtration spin columns (Ambion) according to manufacturer instructions to remove unincorporated nucleotides. Probe concentration was detemined by Qubit RNA assay (Life Technologies) and verified by Agilent Bioanalyzer.

### Hybridization and capture

To ensure even pooling of input libraries, inline barcoded Illumina libraries were pooled equally according to their concentrations as determined by Agilent Bioanalyzer and KAPA real time PCR (KAPA Biosystems) depending on bisulfite (12-plex) or standard sequencing (24-plex) to a total of 1 or 2 ug of library, respectively. Hybridization reactions were assembled similarly to a previous report [5], except hybridization components were scaled up accordingly for final volumes of 52 ul or 104 ul for 12 or 24-plex hybridization reactions, respectively. Library pools were mixed with 40-fold human Cot-1 DNA (Life Technologies), and concentrated to a 15 or 30 ul volume by SpeedVac (Thermo). To determine probe quantity for select targets in individual hybridizations, the theoretical mass yield of target was calculated in picograms based on total BAC target size and male human diploid genome size. A 2500-fold probe∶theoretical target yield mass ratio for individual targets typically yielded between 75–80% of aligned reads within target regions. Probe was brought to a final volume of 10 or 20 ul in nuclease-free water depending on final reaction volume, and 1–2 ul of SUPERase-In (Life Technologies) added for final working probe solution. For hybridization assembly, 26 ul or 52 ul 2× hybridization buffer (10× SSPE, 10× Denhardt's, 10 mM EDTA, and 0.2% SDS) volumes were preheated in 0.5 mL self-standing tubes with screwcaps containing O-rings (USA Scientific) within a hybridization oven. Library pools containing Cot-1 DNA were heated at 95°C for 5 min and then held at 65°C for 5 min on a thermal cycler before they were added to pre-heated hybridization buffer, followed by addition of probe solution preheated to 65°C for 2 min. All assembled hybridizations reactions were incubated at 65°C for 24–60 h within a hybridization oven.

For capture procedure per each 52 ul hybridization reaction, 35 ul of input Dynabeads MyOne Streptavidin C1 were used and washed three times according to manufacturer instructions with binding and wash buffer (Life Technologies). Beads were resuspended in 200 ul bead binding and wash buffer and hybridization reactions are added to the beads and incubated for 30 min with frequent pulse vortexing. After binding, beads were washed twice at room temperature in 0.5 mL hybridization wash buffer 1 (1×SSC, 0.1% SDS), followed by four wash steps at 65°C in preheated 0.5 mL hybridization wash buffer 2 (0.1×SSC, 0.1%SDS) using a heat block and a magnet for 1.7 mL microfuge tubes.

For standard sequencing, washed Dynabeads were resuspended in 0.1M NaOH and pulse vortexed periodically for 10 min to elute captured Illumina library. Eluted library was removed to a new tube containing 70 ul of 1M Tris-HCl pH 7.5 to neutralize the solution, and followed by a SPRI bead cleanup with two 0.5 mL 70% ethanol washes. SPRI beads were set for 10 min and library was eluted in 35 ul of nuclease free water. For bisulfite sequencing, washed Dynabeads were resuspended in 40 ul of EB buffer and transferred into 96 well PCR plates for bisulfite conversion with the Epitect Bisulfite Kit (QIAGEN) according to handbook protocol (conversion of unmethylated cytosines in small amounts of fragmented DNA). Before cleanup, beads were removed from the conversion reaction. Library was eluted twice with 20 ul each of preheated EB buffer.

Final captured libraries were amplified by PCR using 0.5 ul of each standard Illumina primer (25 uM each), 5 ul 5M Betaine (Sigma), 2.5 ul 10 mM dNTP mix (New England Biolabs), 5 ul 10× PCR buffer, 2 ul 50 mM MgCl_2_, 1 ul Platinum Taq, library, and water up to a 50 ul volume. Cycling conditions were 98°C for 1 min, followed by 18–22 cycles (depending on target size) of 95°C for 30 s and 62°C for 3 min 30 s. PCR amplified libraries were cleaned up by SPRI beads. Library concentrations were determined by Agilent Bioanalyzer High Sensitivity DNA assay and real time PCR with a library quantification kit (KAPA Biosystems). For further multiplexing of libraries post-hybridization or post-bisulfite conversion, indexed primers were used in the PCR amplification step, and concentrations of final library pooled sets were determined by an additional real time PCR reaction. Final Illumina library sequencing was performed according to standard protocol using a variety of Illumina sequencer platforms over the course of 3 years (see Table S1).

### Alignment and mapping

Pass filter sequence linked to each index and inline barcodes was demuxed from fastq files and assigned to individual libraries using software that was also used to design inline barcoded adapter sequences. Bordering adapter sequences were removed from reads using the AdapterRemoval software [15]. Standard sequencing fastq files were aligned with BWA in paired end format to the human hg19 reference genome. PCR duplicates were removed and sam files were filtered by q20 mapping quality. For determination of CNV boundaries, read depth was extracted from wig files in non-overlapping 100 bp segments across the length of the target genomic coordinates and the fraction of total bases per segment was calculated. LogR values were determined across the target site by log_2_ of individual cases read fraction divided by the median read fraction of all control samples. For determination of CpG methylation across a target region, human hg19 reference forward and reverse strand sequences were each bisulfite converted in silico and a reference for mapping was built with Bismark. In conjunction with Bowtie2, Bismark was modified to function with local mode for read mapping. Duplicate reads were removed, and methylation values were extracted using Bismark. CpGs with less than a 20× minimum depth coverage were filtered and percent methylation values were used for further data analysis. Alignment files demonstrating coverage of one chr11 target by CATCH-Seq in comparison to WGS from 15 merged individuals from 1000 genomes data within this same target region, in addition to our CATCH-Seq repeat blocking analysis on another chr11 target have been submitted and are available from the National Center for Biotechnology Information (NCBI) Sequence Read Archive (SRA) with BioProject accession number [SRP042633].

## Results and Discussion

### Hybrid selection method

**Figure 1:**
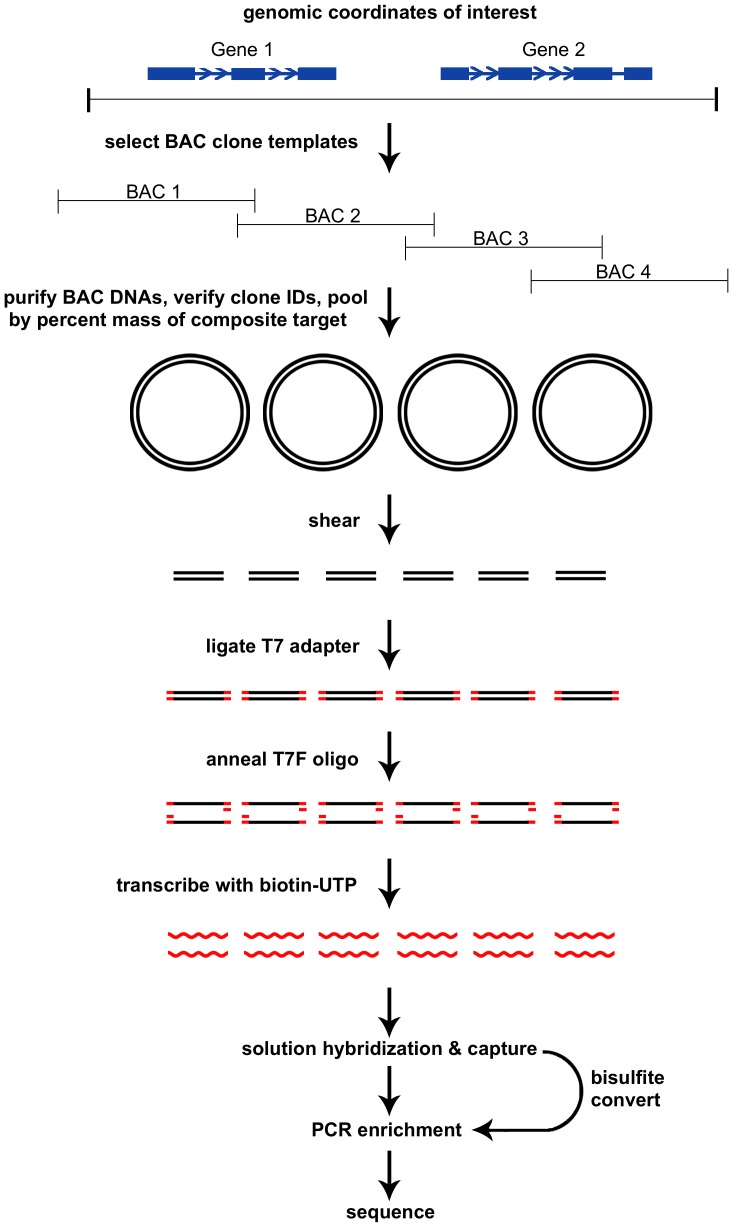
Overview of the clone adapted template capture hybridization sequencing procedure. BAC clone templates are selected to span genomic coordinates of interest, and pooled by percent mass of the composite target. BACs are sheared, ligated with T7 adapters to transcribe biotinylated RNA probes, and then solution hybridized with prepared libraries. Following capture, libraries are amplified by PCR, or bisulfite converted prior to amplification for analysis of DNA methylation. Target enriched libraries are pooled and sequenced.

We developed a simple approach for solution hybridization capture sequencing of large genomic targets without the need for oligonucleotide synthesis of target templates (Figure 1). BAC clones were selected across genomic coordinates of interest to generate templates for probe synthesis. PCR or restriction digest was used to first correctly identify selected clones, and BAC DNA was purified. For composite targets of interest greater than what was covered by a single clone, multiple BAC DNAs (contiguous or discontiguous regions) were pooled based on individual percent of composite size in basepairs of the target multiplied by the mass of input template DNA (Figure 1). Pooled BAC template DNA was randomly sheared, ligated with T7 promoter-containing adapters, and T7 forward adapter oligo was annealed to generate double stranded promoter regions with single stranded antisense templates. Following cleanup of the annealing reaction, in vitro transcription was used in the presence of biotin-UTP to synthesize the probes. A similar approach for probe synthesis was also recently described to enrich for ancient human DNA from environmental contaminants using the entire human genome as a template using T7-promoter containing adapters [18]. Solution hybridization and capture procedures were described previously for Illumina libraries, and our protocol is similar except with increased Cot-1 concentration, larger reaction volume, and additional wash steps [5]. We also typically hybridized library samples in 12-plex or 24-plex using inline barcoded adapters, and adjusted hybridization reaction volumes for scaling up concentration of pooled library and hybridization reagents appropriately. Before PCR enrichment of captured library, bisulfite conversion was also used to analyze DNA methylation in regions of interest by use of conversion-resistant, inline barcoded adapters used in library construction.

Our materials and methods described here represent our most current procedures with all quality assurance steps. Compared to previous methods used for solution-based enrichment using BAC DNA, our procedure does not require nick translation of whole BAC DNA and a 6 h pre-hybridization blocking of the BAC probe [16]. Our method also resembles exome capture methods at the step of probe synthesis and hybridization [5]. Random shearing of the BAC templates and use of in vitro transcription typically yielded a 2–4 fold mass of probe relative to input BAC DNA with probe size ranging from 200–400 bp. Although we do not perform a PCR amplification step after ligation of T7 adapters, addition of this step may provide an improvement in probe yield and vastly reduce the amount of input template DNA required. This template amplification step was used to amplify and incorporate T7-promoter into probe bait templates generated from oligos prior to in vitro transcription, and the biotinylated RNA probe yield was 10–20 fold relative to input template [5]. Overall, our method is designed specifically for next-generation sequencing without oligo synthesis.

### Regional capture and coverage

The percentage of uniquely mapped reads that align to captured target regions provides a metric for the specificity and efficiency of the capture method. We captured 9 independent human genomic targets of varying sizes and sample number ranges across various Illumina sequencing platforms. The mean percentage across samples of uniquely mapped reads that aligned to these target sequences ranged from ∼42% to 82% when considering percentage of mapped reads with a threshold MAPQ of greater than or equal to 20 (Table S1). We found no greater than a 5% reduction in mapped reads when applying this mapping quality filter, and we consistently achieved 80–90% mapping rate of total reads depending on the target region. Our estimated PCR duplication rate ranged between 5 and 40% of sequence per sample, and was influenced by target size, the level of sample plexing within the hybridization, and whether final captures were bisulfite converted. The vast majority of samples were run in 24-plex in a single HiSeq 2000 sequencing lane. As expected, the mean target coverage varied by composite target sizes, read length, and Illumina sequencing platform. All captured targets revealed high enrichment of reads positioned directly within the boundaries of each BAC template selected for probe synthesis (Figure S1). With closer inspection of individual targets, we found a variety of subregions where coverage was sparser and uneven due to large repeats, or repeats with low divergence (Figure S2).

**Figure 2:**
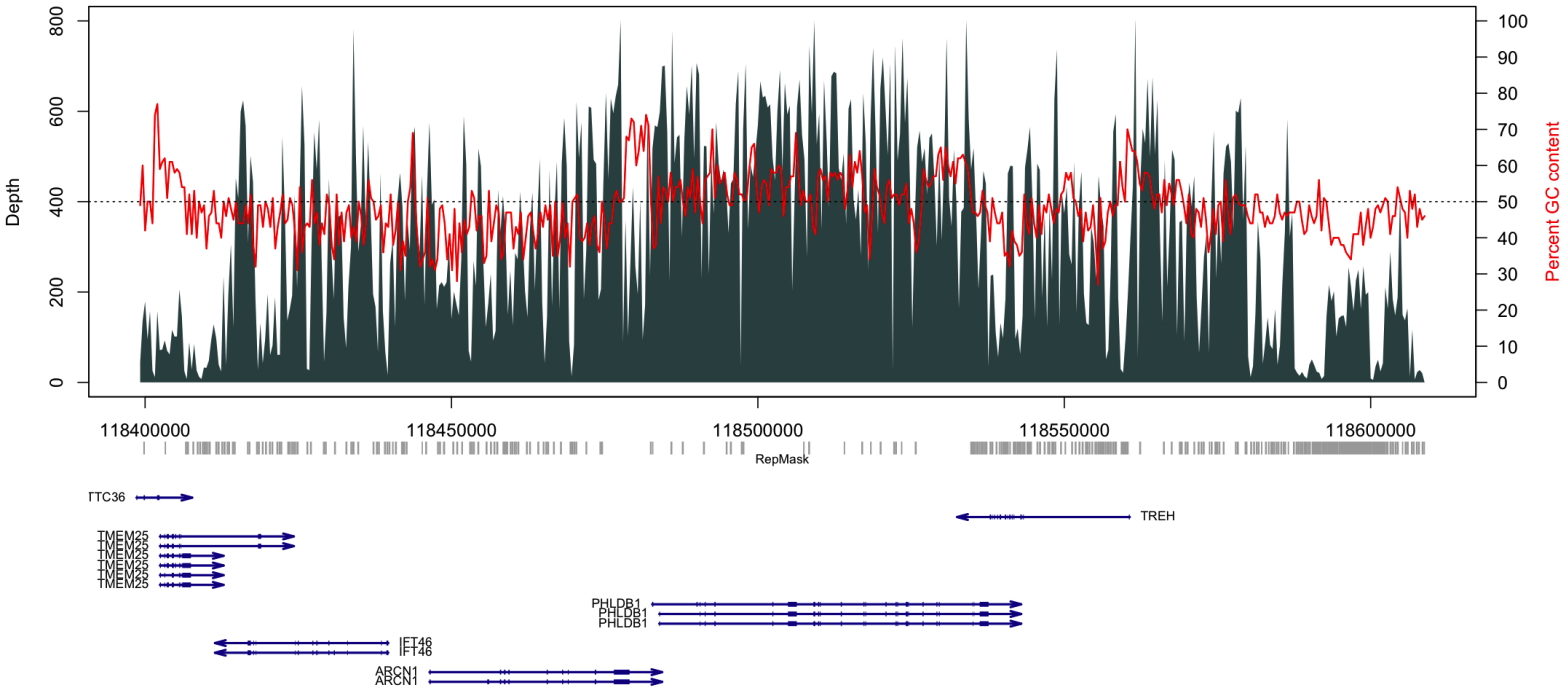
Read depth plot of a chromosome 11 target for a sample showing median coverage among all samples used for capture. Vertical bars indicate read depth with scale depicted on the left side of the panel. Red lines show percent GC content across non-overlapping 400 bp intervals spanning the target region with scale shown on the right side of the panel. Horizontal dotted line indicates 50% GC content. A repeat structure track (RepMask) is shown below the plot in gray derived from the UCSC genome browser for all repeats containing a Smith-Waterman score of at least 600, and larger than 200 bp in size. Genes are shown below the repeat track in dark blue and arrows depict gene orientation.

**Figure 3:**
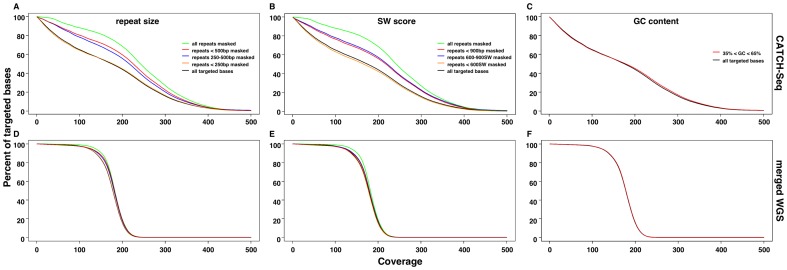
Capture efficiency in a sample representing the median coverage among all sequenced samples shown by the percent of total targeted bases covered at particular coverage depths in a chromosome 11 target. (A) Percent of targeted bases covered using various thresholds of repeat masking (A) by size, or (B) (SW) scores. (C) Percent of targeted bases covered based on masking of percent GC content extremes. Upper panels show coverage by CATCH-Seq within a sample that showed median coverage among all other samples used in the capture. (D–F) Lower panels show coverage within the corresponding captured region for the same number of merged reads analyzed for CATCH-Seq under the same repeat masking or percent GC content thresholds from 15 individuals sequenced for the 1000 genomes project (merged WGS).

Blocking repetitive sites is crucial for solution hybridization based capture systems, as the inclusion of repetitive sequences in a capture probe set can lead to contamination of the final sequence reads with off-target repeats [5]. For CATCH-Seq, blocking of repeats is essential because many of probes synthesized from BAC templates contain repeat regions. Based on our weaker coverage of repeats, we were interested in how both the levels of repeat divergence and repeat size influence enrichment and uniformity of coverage within target template regions. Typical commercial platforms avoid synthesis of probes within repeat regions, and usually only consider uniformity of coverage within non-repetitive sites. We were interested in the uniformity of coverage of both repetitive and non-repetitive sequences as repeats represent a considerable proportion of the contiguous regions we targeted. We specifically analyzed a region on chromosome 11 that is one target within a composite capture of ten targets and selected the sample that represented median coverage among all of the samples we sequenced (Figure 2, Table 1). To understand the influence of repeat structures on target capture uniformity, we compared the base coverage across all targeted bases within our chromosome 11 site after repeat masking the target with increasing threshold values of repeat lengths or Smith-Waterman (SW) scores (Table 1). The variation in repeat masking thresholds gave us an indication of the proportion of our captured sequences that were uniquely mapping to repetitive versus non-repetitive regions within the target site. A mask of repeat sizes below 250 bp in length from target coverage calculations did not proportionally alter coverage rates compared to the total unmasked target, suggesting that small repeats were covered effectively. Repeat mask of sizes ranging between 250 bp and 500 bp increased our relative coverage rate, and was similar to the effect of masking all repeats less than 500 bp (Figure 3A). Masking of all repeats, regardless of size, demonstrated that non-repetitive sequences represented the majority of our capture, and indicated that our blocking approach was highly effective. Repeat masking by SW scores produced a similar trend as repeat size. Capture of repeats above a score of 600 became less efficient (Figure 3B). Extremes in GC content are also known to influence coverage of targeted bases in solution hybridization-based exon capture platforms [6]. We also masked coverage by GC content extremes in 400 bp intervals containing high (>65%) and low GC (<35%) percentages (Figure 2, Table 1). Masking stretches of extreme GC percentages did not alter the relative coverage rate (Figure 3C).

**Table 1:** Repeat structure description within a chromosome 11 target.

repeat ranges	size (kb)	on target (%)^a^
total^b^	112.2	53.6
<250 bp	29.1	13.9
250 bp to 500 bp	58.8	28.1
>500 bp	24.1	11.5
<600SW	14.1	6.8
600SW to 900SW	74.9	35.8
>900SW	23.2	11.1
GC extremes^c^	18.4	8.8

a total captured target size is 209.2 kb, target region shown in Figure 2.

b all repeat hg19 coordinates, sizes, and Smith-Waterman (SW) scores obtained from RepeatMasker within the UCSC Genome Browser. For descriptions of RepeatMasker, http://www.repeatmasker.org/.

c total sequence within the target coordinates with 400 bp intervals containing less than 35% and greater than 65% GC percentage.

We were interested in discerning the difference between blocking of repeats in the hybridization step versus the ability to uniquely map reads within these intermediate and large repeat structures within our chromosome 11 target. We compared our target coverage with the coverage of the same region from whole genome sequencing (WGS) data. Such a comparison should reveal the effects of poor unique mapping within these target repeat regions versus capture bias produced from blocking. We used the merged WGS data of 15 individuals from the 1000 genomes project to approximate the total sequence depth of our capture experiment. We analyzed reads with a MAPQ greater than or equal to 20, and found that repeat masking WGS data made little difference in coverage rate in this same target site (Figure 3E–F). This suggests that repeat blocking within the solution hybridization step has a much greater impact on coverage of target repeat structures than difficulty in uniquely mapping captured reads within these target reference repetitive sequences. The cumulative coverage plots reveal a gradual slope of coverage rates in the target region, indicating a wider range of sequence depths compared to the more uniform coverage of WGS. However, much of this difference can be attributed to repeat blocking. Completely repeat masked coverage calculations within this target between CATCH-Seq and WGS showed very similar numbers of bases covered at 50× depth. CATCH-Seq yielded 89% of targeted bases covered at 100×, compared to 98% for WGS. Alignment files comparing CATCH-Seq and WGS can be found at NCBI SRA with BioProject accession SRP042633.

**Figure 4:**
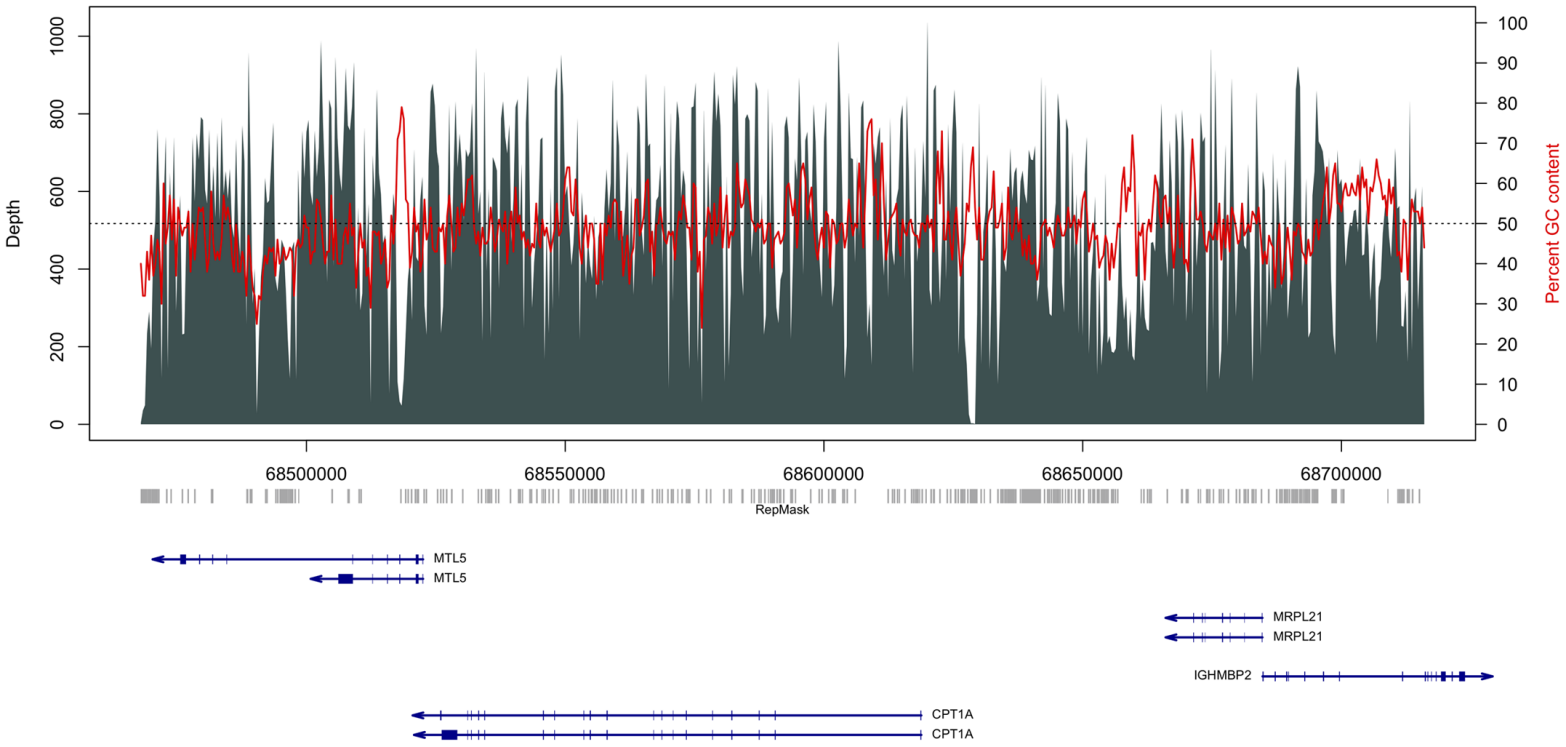
Read depth plot of a chromosome 11 target for a sample showing median coverage among all samples used for capture and bisulfite sequencing. Vertical bars indicate read depth with scale depicted on the left side of the panel. Red lines show percent GC content across non-overlapping 400 bp intervals spanning the target region with scale shown on the right side of the panel. Horizontal dotted line indicates 50% GC content. A repeat structure track (RepMask) is shown below the plot in gray derived from the UCSC genome browser for all repeats containing a Smith-Waterman score of at least 600, and larger than 200 bp in size. Genes are shown below the repeat track in dark blue and arrows depict gene orientation.

**Figure 5:**
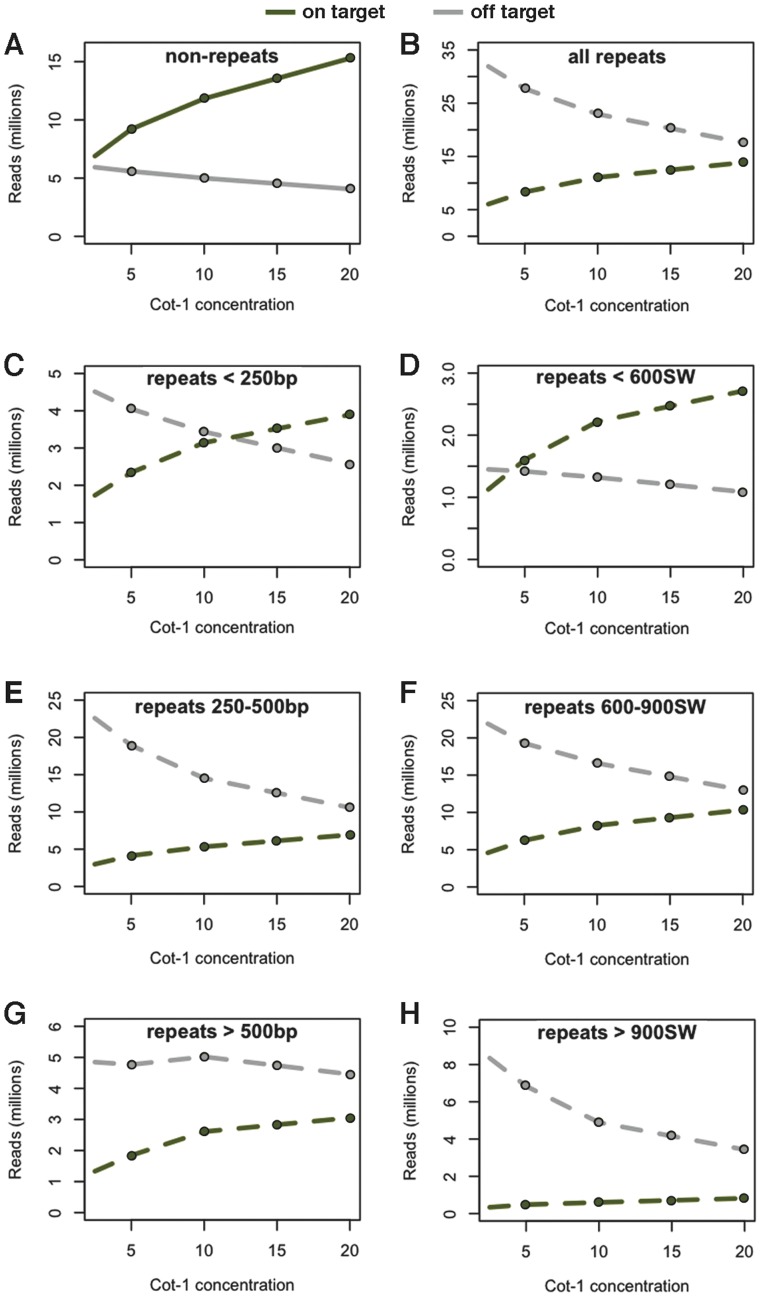
The effect of repeat blocking with increased concentrations of Cot-1 DNA within the CATCH-Seq hybridization step of a chromosome 11 target. Total numbers of on target and off target read yields in millions within non-repetitive sequences (A) or repetitive sequences (B). (C–H) On and off target read yields within repeat structures based on different thresholds of size (C,E,G) or divergence (D,F,H). Green and gray lines show on target and off target reads, respectively.

To further investigate the effect of repeat blocking on target coverage, we performed solution hybridization reactions with libraries prepared from K562 cell line genomic DNA and increasing Cot-1 DNA concentrations to test the influence of this blocking reagent on repeat coverage in another chromosome 11 target that was also captured for bisulfite sequencing of an independent sample set (Table S1, Figure 4). We typically used a 20∶1 concentration of Cot-1 to library ratio and were interested in how reduction of Cot-1 DNA influenced on-target capture in both repeats and non-repeats. Approximately 50 million reads were sampled with a MAPQ of greater than or equal to 20 from the total yield of aligned reads from each hybridization with 2.5, 5, 10, and 20 fold Cot-1 to library ratio. We found that with lower input concentration of Cot-1 DNA, we compromised overall target capture efficiency in both non-repetitive sites and in repeats. Increasing concentrations of Cot-1 DNA improved both the absolute yield of reads on-target while also decreasing off-target yields (Figure 5). CATCH-Seq procedures with no Cot-1 DNA in the hybridization step yielded less than 9% of mapped reads within a target sites. In reads aligned to non-repeat sequence, we found a stronger relationship between Cot-1 concentrations and increasing on-target reads than with reduced off-target reads (Figure 5A). By comparison, increasing Cot-1 concentration produced a roughly equal exchange of reads aligned to off-target repeats as for those aligned to on-target repeats (Figure 5B). We found that this rate of exchange between off-target and on-target repeats varied depending on repeat size and SW score. Smaller repeats of less than 250 bp exhibited an equal exchange in off-target for on-target reads, while larger repeats and those with higher SW scores showed a mild increase in yield of on-target reads, while off-target yields declined (Figure 5C–H). Overall, the highest Cot-1 concentration at 20 fold produced the highest on-target read yield.

We observed that absolute on-target read yields were split almost evenly between repeats and non-repeat regions at all Cot-1 concentrations. Therefore, we expressed read yields as a percentage of total yield stratified by non-repeats, repeat size, or SW score across each hybridization experiment with increasing Cot-1 concentration (Figure S3). The largest percent increase of on-target reads was within non-repeats, and the largest percent decrease was in intermediate sized repeats or SW scores, while yields within the smallest and largest repeats changed very little relative to the total yield. We found that the overall percent yield proportion of on-target reads alone to one another within classified categories did not vary with Cot-1 concentrations, and these percentages of on-target reads also proportionally represented the overall repeat and non-repeat structure within this specific target site (Table 2). We did find about a 5% bias of on-target yield toward non-repeats when comparing to total percent repeats across this target site (52.4% of target bases are repeats with 47.6% of read yield, and 47.6% of target bases are non-repeats with 52.4% of read yield). These results suggest that the target sequence yield proportionally represents the diversity of the target region itself. Alignment files comparing CATCH-Seq with increasing concentrations of Cot-1 can be found at NCBI SRA with BioProject accession SRP042633.

**Table 2:** Repeat structure description within a chromosome 11 target used for Cot-1 tests.

repeat ranges	size (kb)	target bases (%)^a^	on target reads (%)^b^
total^c^	129.7	52.4	47.6
<250 bp	32.0	12.9	13.4
250 bp to 500 bp	72.4	29.2	23.7
>500 bp	25.2	10.2	10.5
<600SW	19.6	7.9	9.3
600SW to 900SW	97.4	39.3	35.4
>900SW	12.6	5.1	2.8
non-repeats	118.0	47.6	52.4

a total captured target size is 247.6 kb; target region shown in Figure 4.

b out of 50 million sampled reads at 20× Cot-1 concentration.

c all repeat hg19 coordinates, sizes, and Smith-Waterman (SW) scores obtained from RepeatMasker within the UCSC Genome Browser. For descriptions of RepeatMasker, http://www.repeatmasker.org/.

We also found that the PCR duplication rate in our sequencing reads also increased with Cot-1 concentration. However, we have found that the duplication rate was more strongly influenced by input library quality, and this problem is not unique to our capture platform. Measurement of the true library concentration by qPCR is critical to ensure proper library input into the hybridization for higher diversity of unique reads mapped. Our current procedure uses 40× Cot-1 DNA and with higher quality library (10–40% ligated fragments) one should expect between 5–20% duplication rate, depending on composite target size, hybridization multiplexing, and if the captured library was bisulfite converted. Based on our results, we believe the benefit of high specificity and enrichment within a target region outweighs the compromise in consequential mapped read diversity and lower coverages within intermediate to large repeat regions. Furthermore, default design conditions for commercial platforms avoid the synthesis of probes within repeat regions that would typically not be covered. We found that CATCH-Seq adequately covered all of the same sites where prospective probes would be synthesized based on commercial design within one of our targets (Figure S4). Comparison to WGS coverage from 15 lanes of sequencing yielded better uniformity across repeats, but uniformity outside of repeats was similar. These data show that CATCH-Seq has the ability to capture the same prospective target regions as commercial systems even with repeats included in the probe synthesis. Furthermore, while no custom capture platform will prospectively be able to cover as uniformly as WGS as a general caveat of capture sequencing, samples can be highly multiplexed and still achieve higher coverage per individual within a target compared to 15 lanes of WGS on a single sample.

### Applications of CATCH-Seq

**Figure 6:**
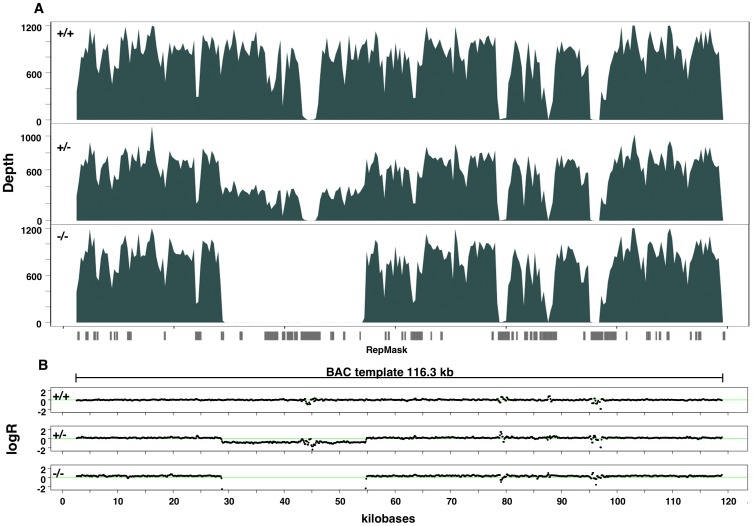
Determination of copy number variation across a CATCH-Seq target using read depth. (A) Read depths are partitioned into 100 bp segments across the length of target genomic coordinates and the fraction of total aligned bases per segment are calculated. In this target, there is a noticeable drop in read depth in two individuals shown in bottom panels compared to wild type (+/+) that indicates individuals that contain heterozygous (+/−) and homozygous (−/−) deletions in this region. (B) Log-ratio values (logR) are calculated across the target site that are normalized for read depth variance caused by capture and sequencer biases to resolve clear copy number variation boundaries. Contained within the deleted region is a repeat sequence as shown by underlying RepeatMasker track (RepMask) that is not well covered. Coverage of this repeat structure is reflected in the logR plot as a slight fluctuation from zero as indicated by the horizontal green lines. For targets containing a copy number variation that represents a large proportion of the total target sequence such as the one depicted here, often the individual base fraction normalization by the median of control samples will result in slightly elevated logR values outside the variable region that is most noticeable in the individual containing the homozygous deletion in the bottom panel. The extent of the BAC template used for CATCH-Seq is depicted just below the RepMask track.

Based on our sequence enrichment procedures and our kilobase scale contiguous probe sets, we have found this technique useful for distinguishing both genetic and epigenetic variation across different target sites. Besides variant calling within target regions, we have found that the level of specificity and unique high coverage across targets allows for the resolution of large CNV boundaries (Figure 6A). By partitioning of the total reads into 100 bp segments across the length of target genomic coordinates and determining the fraction of total aligned bases per segment, we are able to calculate log-ratio values across the target site that normalize read depth variance caused by capture and sequencer biases. We calculate the log_2_ of an individual's base fraction within a segment divided by the median base fraction of all control samples. With the advantage of the normalized read depth, we are able to identify both homozygous and heterozygous duplications and deletions within target sites (Figure 6B). These data show that even with varying levels of coverage across the site, we are able to effectively resolve CNVs. The majority of targets containing CNVs were pre-selected based on results from high-density genotyping arrays that implicated a CNV within the specific locus. Recent studies have shown that CNVs such as inversions, deletions, insertions, and segmental duplications may contribute another level of genetic variation that may influence human phenotypic diversity and is also associated with a variety of human diseases [19], [20]. Often the most common approach of CNV determination involves SNP arrays, but often arrays cannot clearly establish CNV boundaries [21]. Our method provides a unique validation to resolve CNV boundaries.

**Figure 7:**
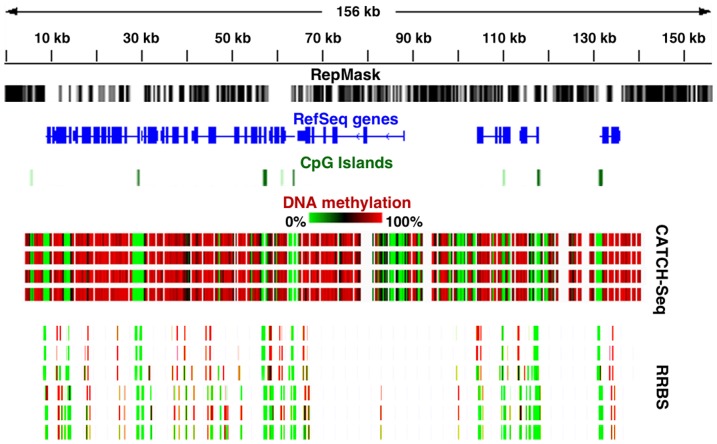
High density methylation data derived from bisulfite sequencing of a CATCH-Seq target. Scale of the captured region is indicated in the topmost track in kilobases (kb), followed by repeat structure in gray and black (RepMask), genes shown in blue (RefSeq), and CpG islands in green. Four CATCH-Seq tracks from the same cell type show DNA methylation levels across ∼2,700 target CpGs with hypomethylation depicted in green and hypermethylation in red. Six reduced representation bisulfite sequencing (RRBS) tracks for different cell and tissue types correspond with the same captured region, and demonstrate CpGs not covered by RRBS method compared to CATCH-Seq. The four CATCH-Seq tracks are from the same cell type as the topmost RRBS track. RRBS tracks are derived from previously reported data [23]. CpGs shown within CpG islands were all typically hypomethylated across all cell and tissue types depicted.

We have also applied CATCH-Seq to analysis of DNA methylation across large target sites containing CpG islands and multiple genes. By using methylated adapters and treating the post-capture libraries with bisulfite conversion, we can measure CpG methylation with higher coverage thresholds than what is often generated for genome-wide analysis of individual CpGs such as by reduced representation bisulfite sequencing (RRBS) (Figure 7). Furthermore, we have found that CATCH-Seq can also be used in parallel with any other practiced functional genomics approaches. A similar method to ours performed sequencing of BAC-enriched mononucleosomal fragments (known as BEM-Seq), using whole BAC labelled probes for capture of MNAse-digested fragments within a target site [17]. We have similarly used CATCH-Seq procedures with MNase-digests also from sorted mouse lymphocytes in combination with methylation analysis (unpublished). Lastly, CATCH-Seq was also used to capture gene regions associated with melanism from genomes of unsequenced Felid species using selected fosmids from *Felis catus* as templates [22] (manuscript submitted). Overall, we find that data from CATCH-Seq procedures allows for affordable, high resolution sequencing of captured genomic targets without the added cost of oligo-based probe synthesis. We have included a price per sample estimation with comparison of current commercially available custom probe synthesis platforms for two of our targets we captured (Table S2).

## Supporting Information

Figure S1 **Read depth plots of various BAC-template enriched sequencing reads as shown within the UCSC genome browser.** Table 1 provides further details of each target depicted for which human hg19 genomic coordinates are shown above each individual target. From top to bottom black vertical lines indicate sequencing read depth, followed by genes contained within the target, and selected BACs used as templates.
(TIFF)

Figure S2 **Zoomed CATCH-Seq targets shown within the UCSC genome browser that exhibit low read depth covering repetitive sites.** Black vertical lines show read depth; light gray to black tracks below indicate repeat sequences with darker shades indicating lower divergence or higher similarity to other repeats across the genome.
(TIFF)

Figure S3 **On and off target read yields expressed as a percentage of total yield stratified by non-repeats, repeat size, or SW score across each hybridization experiment that contained increased concentrations of Cot-1 DNA.** On and off target read yield percentages according to repeat size thresholds (A) or by Smith-Waterman (SW) repeat scores (B). Black and shades of gray show off target reads; white and shades of green depict on target reads.
(TIFF)

Figure S4 **A zoomed in chr11 region within the UCSC genome browser (as depicted in** **Figure 2** **) showing read depth of CATCH-Seq compared to WGS that contains many SINE elements.** The majority of unevenness across the capture is found within SINE repeats. Another track depicts prospective probe baits recommended for synthesis using default parameters with NimbleDesign software for custom capture sequencing where probe is completely repeat masked. CATCH-Seq effectively covers the exact sites where probes are recommended for synthesis.
(TIFF)

Table S1 **CATCH-Seq target capture summary.**
(DOCX)

Table S2 **CATCH-Seq cost per sample comparison estimation summary.**
(DOCX)

## References

[1] Borate U, Absher D, Erba HP, Pasche B (2012) Potential of whole-genome sequencing for determining risk and personalizing therapy: focus on AML. Expert Rev Anticancer Ther 12: 1289–1297. 23176617 10.1586/era.12.116 PMC3636990

[2] Whitcomb DC (2012) What is personalized medicine and what should it replace? Nat Rev Gastroenterol Hepatol 9: 418–424. 22614753 10.1038/nrgastro.2012.100 PMC3684057

[3] Mertes F, Elsharawy A, Sauer S, van Helvoort JM, van der Zaag PJ, et al (2011) Targeted enrichment of genomic DNA regions for next-generation sequencing. Brief Funct Genomics 10: 374–386. 22121152 10.1093/bfgp/elr033 PMC3245553

[4] Goh G, Choi M (2012) Application of whole exome sequencing to identify disease-causing variants in inherited human diseases. Genomics Inform 10: 214–219. 23346032 10.5808/GI.2012.10.4.214 PMC3543920

[5] Gnirke A, Melnikov A, Maguire J, Rogov P, LeProust EM, et al (2009) Solution hybrid selection with ultra-long oligonucleotides for massively parallel targeted sequencing. Nat Biotechnol 27: 182–189. 19182786 10.1038/nbt.1523 PMC2663421

[6] Clark MJ, Chen R, Lam HY, Karczewski KJ, Euskirchen G, et al (2011) Performance comparison of exome DNA sequencing technologies. Nat Biotechnol 29: 908–914. 21947028 10.1038/nbt.1975 PMC4127531

[7] Irizarry RA, Ladd-Acosta C, Wen B, Wu Z, Montano C, et al (2009) The human colon cancer methylome shows similar hypo- and hypermethylation at conserved tissue-specific CpG island shores. Nat Genet 41: 178–186. 19151715 10.1038/ng.298 PMC2729128

[8] Heyn H, Esteller M (2012) DNA methylation profiling in the clinic: applications and challenges. Nat Rev Genet 13: 679–692. 22945394 10.1038/nrg3270

[9] Kaper F, Swamy S, Klotzle B, Munchel S, Cottrell J, et al (2013) Whole-genome haplotyping by dilution, amplification, and sequencing. Proc Natl Acad Sci U S A 110: 5552–5557. 23509297 10.1073/pnas.1218696110 PMC3619281

[10] Porreca GJ, Zhang K, Li JB, Xie B, Austin D, et al (2007) Multiplex amplification of large sets of human exons. Nat Methods 4: 931–936. 17934468 10.1038/nmeth1110

[11] Johansson H, Isaksson M, Sorqvist EF, Roos F, Stenberg J, et al (2011) Targeted resequencing of candidate genes using selector probes. Nucleic Acids Res 39: e8. 21059679 10.1093/nar/gkq1005 PMC3025563

[12] Diep D, Plongthongkum N, Gore A, Fung HL, Shoemaker R, et al (2012) Library-free methylation sequencing with bisulfite padlock probes. Nat Methods 9: 270–272. 22306810 10.1038/nmeth.1871 PMC3461232

[13] Varley KE, Mitra RD (2008) Nested Patch PCR enables highly multiplexed mutation discovery in candidate genes. Genome Res 18: 1844–1850. 18849522 10.1101/gr.078204.108 PMC2577855

[14] DeAngelis MM, Wang DG, Hawkins TL (1995) Solid-phase reversible immobilization for the isolation of PCR products. Nucleic Acids Res 23: 4742–4743. 8524672 10.1093/nar/23.22.4742 PMC307455

[15] Lindgreen S (2012) AdapterRemoval: easy cleaning of next-generation sequencing reads. BMC Res Notes 5: 337. 22748135 10.1186/1756-0500-5-337 PMC3532080

[16] Bashiardes S, Veile R, Helms C, Mardis ER, Bowcock AM, et al (2005) Direct genomic selection. Nat Methods 2: 63–69. 16152676 10.1038/nmeth0105-63

[17] Yigit E, Zhang Q, Xi L, Grilley D, Widom J, et al (2013) High-resolution nucleosome mapping of targeted regions using BAC-based enrichment. Nucleic Acids Res 41: e87. 23413004 10.1093/nar/gkt081 PMC3627574

[18] Carpenter ML, Buenrostro JD, Valdiosera C, Schroeder H, Allentoft ME, et al (2013) Pulling out the 1%: Whole-Genome Capture for the Targeted Enrichment of Ancient DNA Sequencing Libraries. The American Journal of Human Genetics 93: 852–864. 24568772 10.1016/j.ajhg.2013.10.002 PMC3824117

[19] Craddock N, Hurles ME, Cardin N, Pearson RD, Plagnol V, et al (2010) Genome-wide association study of CNVs in 16,000 cases of eight common diseases and 3,000 shared controls. Nature 464: 713–720. 20360734 10.1038/nature08979 PMC2892339

[20] Henrichsen CN, Chaignat E, Reymond A (2009) Copy number variants, diseases and gene expression. Hum Mol Genet 18: R1–8. 19297395 10.1093/hmg/ddp011

[21] Winchester L, Yau C, Ragoussis J (2009) Comparing CNV detection methods for SNP arrays. Brief Funct Genomic Proteomic 8: 353–366. 19737800 10.1093/bfgp/elp017

[22] Schneider A, David VA, Johnson WE, O'Brien SJ, Barsh GS, et al (2012) How the leopard hides its spots: ASIP mutations and melanism in wild cats. PLoS One 7: e50386. 23251368 10.1371/journal.pone.0050386 PMC3520955

[23] Meissner A, Mikkelsen TS, Gu H, Wernig M, Hanna J, et al (2008) Genome-scale DNA methylation maps of pluripotent and differentiated cells. Nature 454: 766–770. 18600261 10.1038/nature07107 PMC2896277

