# Supplementary figures and images for: Targeted Sequencing of Large Genomic Regions with CATCH-Seq
Source: PLoS One. 2014 Oct 30;9(10):e111756. doi: 10.1371/journal.pone.0111756 (PMC4214737; doi:10.1371/journal.pone.0111756)

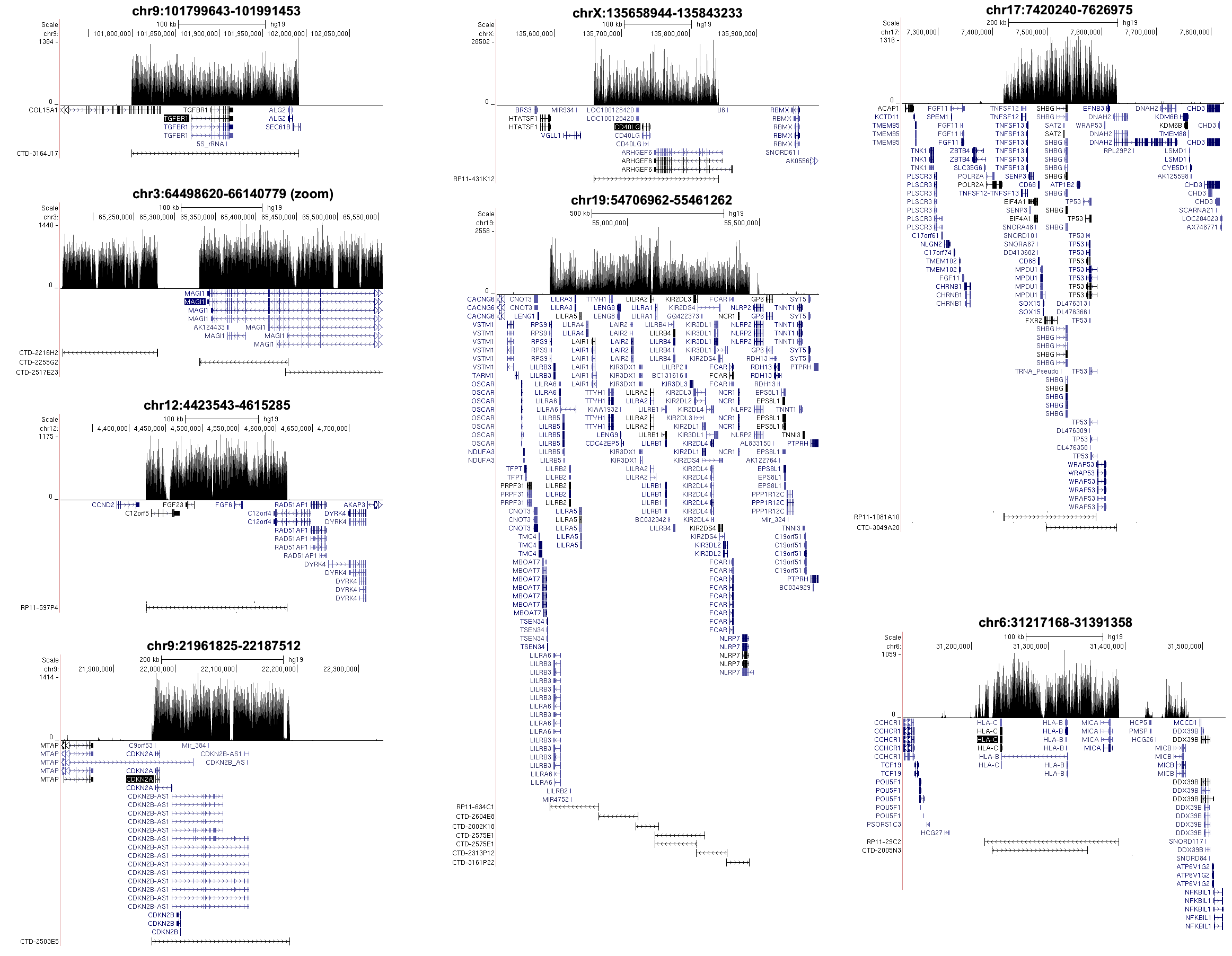

Supplement: Figure S1 — Read depth plots of various BAC-template enriched sequencing reads as shown within the UCSC genome browser. Table 1 provides further details of each target depicted for which human hg19 genomic coordinates are shown above each individual target. From top to bottom black vertical lines indicate sequencing read depth, followed by genes contained within the target, and selected BACs used as templates. (TIFF) [file pone.0111756.s001.tiff]

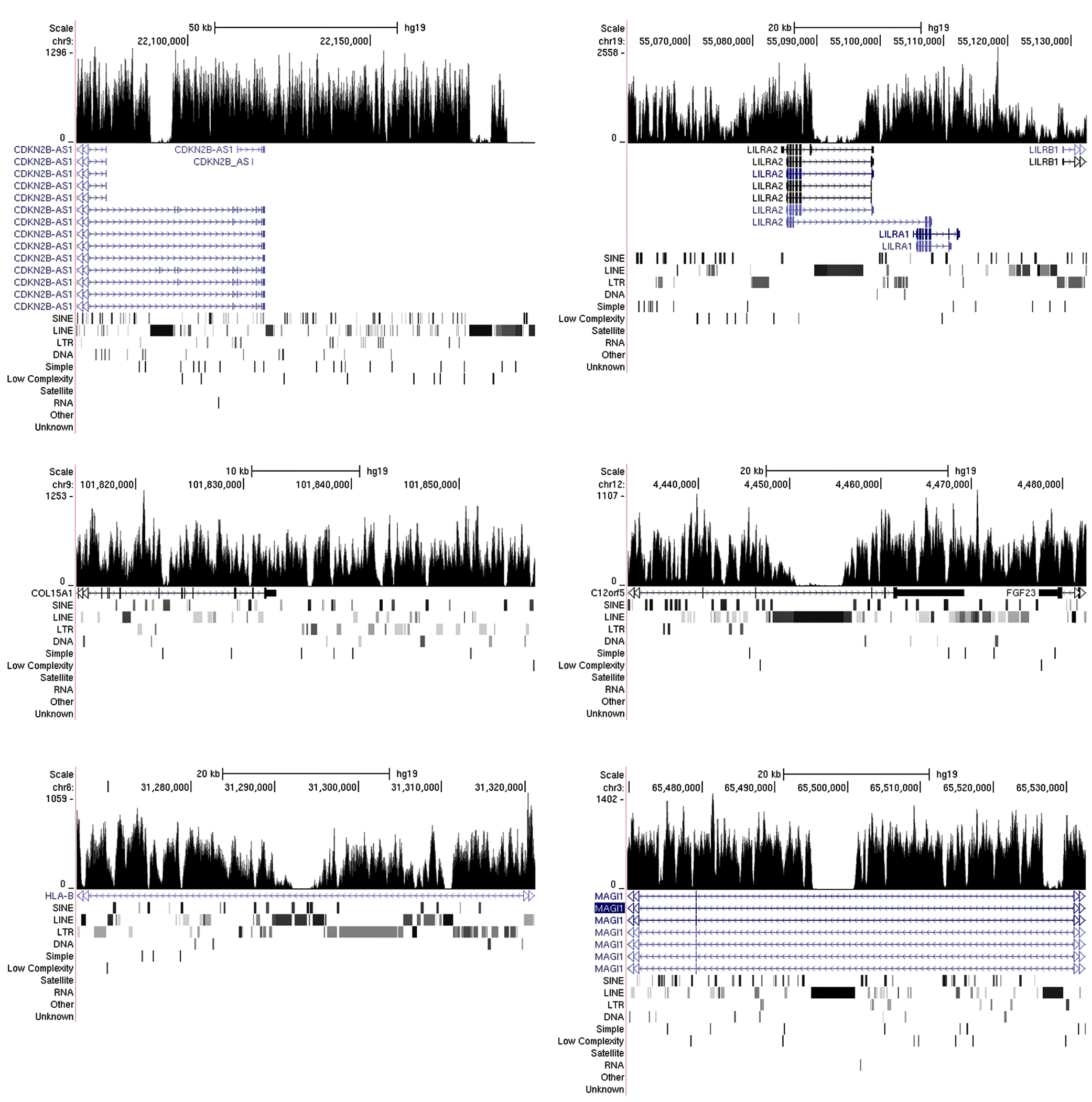

Supplement: Figure S2 — Zoomed CATCH-Seq targets shown within the UCSC genome browser that exhibit low read depth covering repetitive sites. Black vertical lines show read depth; light gray to black tracks below indicate repeat sequences with darker shades indicating lower divergence or higher similarity to other repeats across the genome. (TIFF) [file pone.0111756.s002.tiff]

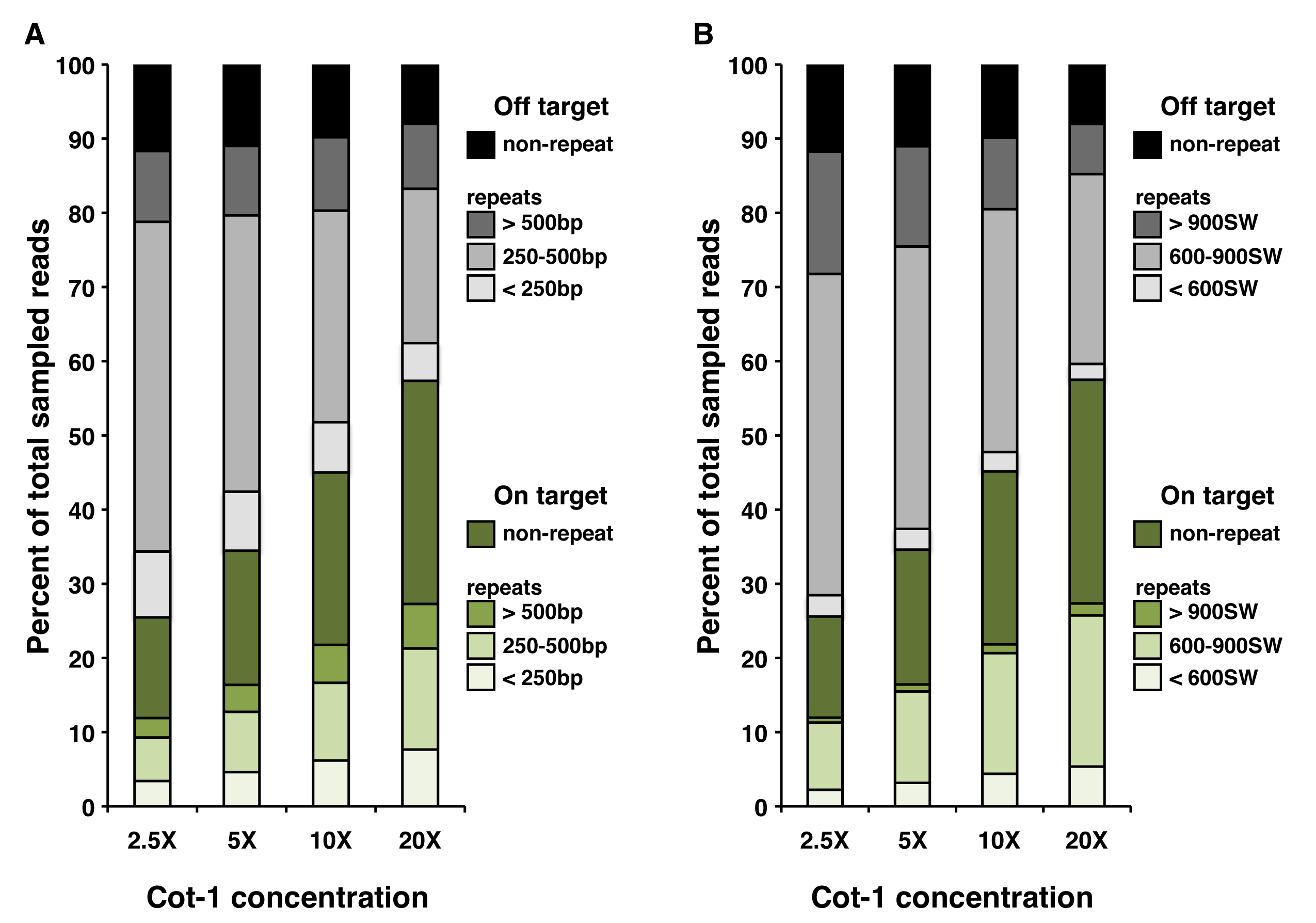

Supplement: Figure S3 — On and off target read yields expressed as a percentage of total yield stratified by non-repeats, repeat size, or SW score across each hybridization experiment that contained increased concentrations of Cot-1 DNA. On and off target read yield percentages according to repeat size thresholds (A) or by Smith-Waterman (SW) repeat scores (B). Black and shades of gray show off target reads; white and shades of green depict on target reads. (TIFF) [file pone.0111756.s003.tiff]

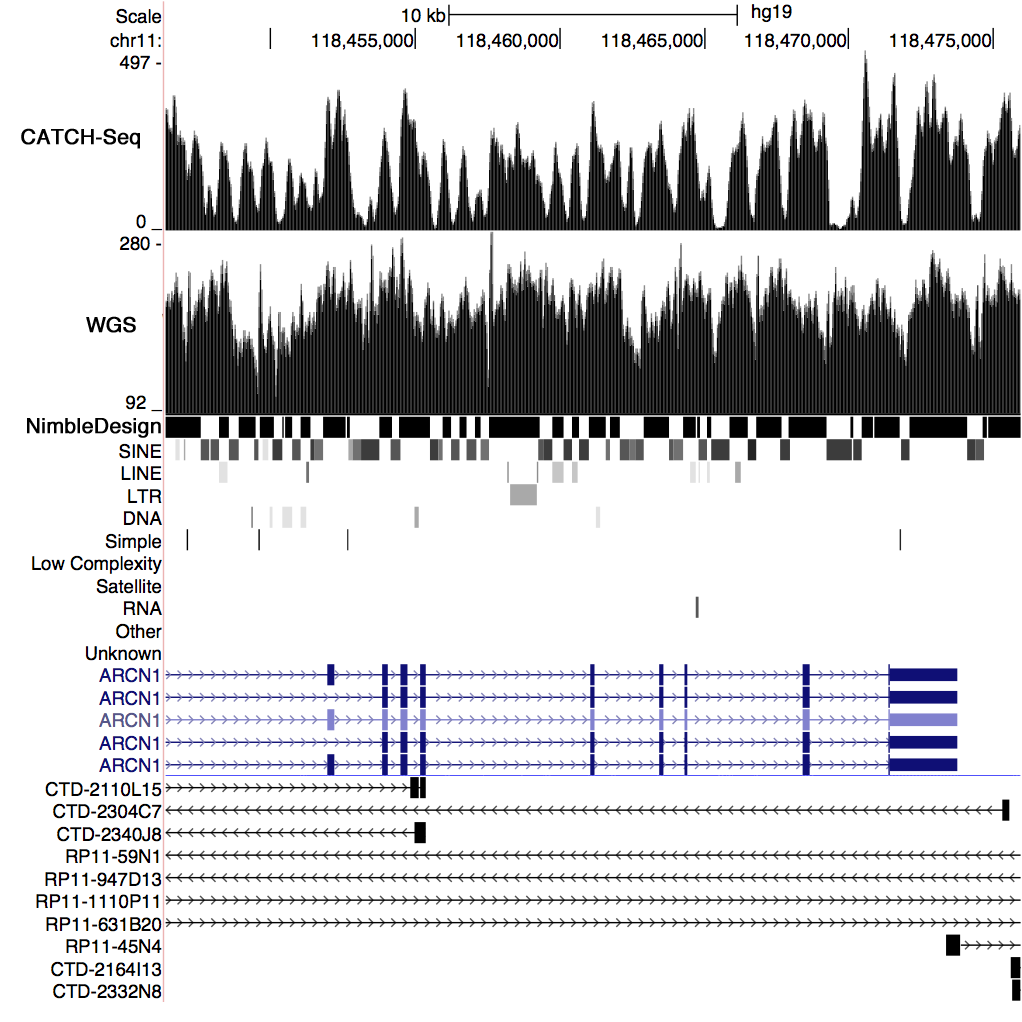

Supplement: Figure S4 — A zoomed in chr11 region within the UCSC genome browser (as depicted in Figure 2 ) showing read depth of CATCH-Seq compared to WGS that contains many SINE elements. The majority of unevenness across the capture is found within SINE repeats. Another track depicts prospective probe baits recommended for synthesis using default parameters with NimbleDesign software for custom capture sequencing where probe is completely repeat masked. CATCH-Seq effectively covers the exact sites where probes are recommended for synthesis. (TIFF) [file pone.0111756.s004.tiff]
