# Supplementary material for: Targeted Sequencing of Large Genomic Regions with CATCH-Seq
Source: PLoS One. 2014 Oct 30;9(10):e111756. doi: 10.1371/journal.pone.0111756 (PMC4214737; doi:10.1371/journal.pone.0111756)
Supplement: Table S1 — CATCH-Seq target capture summary. (DOCX) [file pone.0111756.s005.docx]

| **genomic target** | **size (Mb)** | **hyb plex** | **seq plex** | **samples** | **run** | **total reads^a^** | **aligned reads^b^** | **on target (%)^c^** | **coverage^d^** |
| --- | --- | --- | --- | --- | --- | --- | --- | --- | --- |
| standard sequencing |  |  |  |  |  |  |  |  |  |
| region 1^e^ | 0.192 | 24 | 24 | 48 | GAII 36bp | 1.5 ± 0.7 | 1 ± 0.5 | 79.4 ± 1 | 122 ± 57 |
| region 2^f^ | 1.30 | 4 | 24 | 200 | HiSeq 2x50bp | 7.2 ± 2.8 | 4.5 ± 1.8 | 82.6 ± 10 | 267 ± 105 |
| region 3^g^ | 0.850 | 4 | 24 | 187 | HiSeq 2x50bp | 7.3 ± 2.7 | 4.8 ± 2.0 | 77.4 ± 1.9 | 343 ± 161 |
| region 4^h^ | 0.174 | 4 | 12 | 12 | GAIIx 2x36bp | 2.7 ± 1.2 | 1.0 ± 0.4 | 42.5 ± 4.0 | 146 ± 62 |
| region 5^i^ | 0.259 | 22 | 22 | 22 | HiSeq2.5k 2x150bp | 7.7 ± 3.6 | 2.9 ± 1.4 | 49.5 ± 3 | 1519 ± 825 |
| region 6^j^ | 3.62 | 24 | 24 | 144 | HiSeq 2x100bp | 7.7 ± 4.4 | 3.3 ± 2.1 | 73.1 ± 4 | 130 ± 84 |
| bisulfite sequencing |  |  |  |  |  |  |  |  |  |
| region 7^k^ | 0.226 | 4-12 | 12 | 179 | GAIIx 2x72bp | 2.2 ± 1 | 1.5 ± 0.8 | 47.6 ± 11 | 457 ± 319 |
| region 8^l^ | 0.184 | 11 | 11 | 22 | HiSeq2.5k 2x100bp | 8.2 ± 4.5 | 3.4 ± 2.0 | 40.0 ± 7 | 683 ± 410 |
| region 9^m^ | 0.248 | 12 | 180 | 180 | HiSeq2.5k 2x100bp | 1.2 ± 0.8 | 0.35 ± 0.24 | 56.5 ± 6 | 334 ± 245 |

^a^ average in millions ± standard deviation per individual sample, read pairs indicated for paired end runs

^b^ average in millions ± standard deviation per individual sample, deduplicated, uniquely aligned reads or read pairs, mapq≥20

^c^ (aligned mapq≥q20 reads within target region/total aligned mapq≥20 reads) × 100; per sample

^d^ (aligned mapq≥q20 reads within target region × read length) /total target size in bp per sample; non-repeat masked target

^e^ chr9:101799643-101991453

^f^ chr3:64498620-64676979, chr3:65162637-65278952, chr3:65330695-66140779, chr12:4423543-4615285

^g^ chr19:54706962-55461262, chr12:10535008-10630933

^h^ chr6:31217168-31391358

^i^ chr1:161405089-161663925

^j^ chr2:40290921-40818502, chr5:1207045-1369139, chr7:54912540-55418874, chr8:130269738-130884901, chr9:21867848-22056873, chr10:21747581-22031097,

chr11:118399273-118608438, chr12:128475121-129218891, chr17:7420240-7626975, chr20:62214729-62391570

^k^ chr9 target aligned with Bismark/bowtie; chrX and chr11 targets aligned with Bismark/bowtie2 with mapq≥20, 2 lanes used for each on HiSeq2.5k

^l^ chr9:21961825-22187512

^m^ chrX:135658944-135843233

^n^ chr11:68468357-68715991
