# Supplementary material for: Targeted Sequencing of Large Genomic Regions with CATCH-Seq
Source: PLoS One. 2014 Oct 30;9(10):e111756. doi: 10.1371/journal.pone.0111756 (PMC4214737; doi:10.1371/journal.pone.0111756)
Supplement: Table S2 — CATCH-Seq cost per sample comparison estimation summary. (DOCX) [file pone.0111756.s006.docx]

| **genomic target** | **size (Mb)** | **samples** | **CATCH-Seq cost^a,b^** | **Nimblegen/Roche cost^c^** | **Agilent cost^d^** | **Illumina cost^e^** |
| --- | --- | --- | --- | --- | --- | --- |
| Target 1 (43 BACs)^f^ | 3.62 | 144 | $32.8 | $183.9 | $250 | $173.5 |
| Target 2 (4 BACs)^g^ | 0.248 | 180 | $4.2 | $183.9 | $180 | NA |

^a^ cost estimate per sample considers everything required for preparation of probe baits before hybridization (see Figure 1) as library construction,

sequencing, and costs for subsequent steps will be roughly equal among platforms

^b^ cost for CATCH-Seq includes price for all individual BACs covering the target region, screening, midi/maxi scale purification of BAC DNAs, T7 adapter

ligation, biotin-11-UTP, in vitro transcription reactions and cleanup

^c^ based on price quotation for 96 reactions with probe reagent split for duplex hybridization reactions to cover all samples

^d^ based on price quotation for SureSelect XT 96 reactions with probe reagent split for duplex hybridization reactions to cover all samples

^e^ based on online price quotation for TruSeq Custom Enrichment 96 reactions with probe reagent split for duplex hybridization reactions to cover all

samples;price for Target 2 could not be estimated as the prospective number of probes was below the minimum requirement of 2500 probes

^f^ chr2:40290921-40818502, chr5:1207045-1369139, chr7:54912540-55418874, chr8:130269738-130884901, chr9:21867848-22056873,

chr10:21747581-22031097, chr11:118399273-118608438, chr12:128475121-129218891, chr17:7420240-7626975, chr20:62214729-62391570

^g^ chr11:68468357-68715991
